# Opinions on conscientious objection to induced abortion among Finnish medical and nursing students and professionals

**DOI:** 10.1186/s12910-015-0012-1

**Published:** 2015-03-25

**Authors:** Petteri Nieminen, Saara Lappalainen, Pauliina Ristimäki, Markku Myllykangas, Anne-Mari Mustonen

**Affiliations:** University of Eastern Finland, Faculty of Health Sciences, School of Medicine, Institute of Biomedicine/Anatomy, P.O. Box 1627, FI-70211 Kuopio, Finland; University of Eastern Finland, Faculty of Health Sciences, School of Medicine, Institute of Public Health and Clinical Nutrition, P.O. Box 1627, FI-70211 Kuopio, Finland; University of Eastern Finland, Faculty of Science and Forestry, Department of Biology, P.O. Box 111, FI-80101 Joensuu, Finland

**Keywords:** Conscientious objection, Induced abortion, Reproductive health, Survey

## Abstract

**Background:**

Conscientious objection (CO) to participating in induced abortion is not present in the Finnish health care system or legislation unlike in many other European countries.

**Methods:**

We conducted a questionnaire survey with the 1^st^- and the last-year medical and nursing students and professionals (548 respondents; response rate 66–100%) including several aspects of the abortion process and their relation to CO in 2013.

**Results:**

The male medical respondents chose later time points of pregnancy than the nursing respondents when considering when the embryo/fetus “becomes a person”. Of all respondents, 3.5–14.1% expressed a personal wish to CO. The medical professionals supported the right to CO more often (34.2%) than the nursing professionals (21.4%), while ≥62.4% could work with someone expressing CO. Yet ≥57.9% of the respondents anticipated social problems at work communities caused by CO. Most respondents considered self-reported religious/ethical conviction to be adequate for CO but, at the same time, 30.1–50.7% considered that no conviction would be sufficient. The respondents most commonly included the medical doctor conducting surgical or medical abortion to be eligible to CO. The nursing respondents considered that vacuum suction would be a better justification for CO than medical abortion. The indications most commonly included to potential CO were second-trimester abortions and social reasons. Among the medical respondents, the men were more willing to grant CO also in case of a life-threatening emergency of the pregnant woman.

**Conclusions:**

While the respondents mostly seemed to consider the continuation of adequate services important if CO is introduced, the viewpoint was often focused on the staff and surgical abortion procedure instead of the patients. The issue proved to be complex, which should be taken into consideration for legislation.

**Electronic supplementary material:**

The online version of this article (doi:10.1186/s12910-015-0012-1) contains supplementary material, which is available to authorized users.

## Background

The Finnish health care system is relatively liberal regarding the right for induced abortion until the 12^th^ gestational week [1]. The woman is legally obliged to provide reasons, why the continuation of pregnancy would be a significant burden (*i.e*., so called social indications), but in practice, abortion is allowed virtually for everybody requesting it during this period. After the 12^th^ gestational week, the indications are much more restricted and mostly include serious medical conditions of the woman (gynecological malignancies, *etc*.) or the fetus (neural tube defects, trisomies, *etc*.). Between weeks 20–24, only very serious medical conditions of the fetus (such as trisomy 13) are accepted as indications. After the 12^th^ week, a special application to authorities is required, while before this time point physicians in primary health care can simply refer the woman to an obstetrician/gynecologist (OB/GYN) for abortion.

Induced abortion is a multi-professional process. In primary health care, nurses and general practitioners meet the patient before she is sent to a gynecology unit at a hospital. For surgical abortion, the woman will be taken care by a team consisting of nurses (both at a ward and operating theatre), radiologists, anesthesiologists, midwives and supporting staff, such as ward technicians among others. In case of medical abortion with, *e.g*., mifepristone + misoprostol, at least a nurse, a radiologist and an OB/GYN are required. After the procedure, primary health care meets the woman once more for a follow-up and discussion on contraception. This involves, again, at least a nurse and a general practitioner. In Finland, 87% of the induced abortions are performed medically [2].

Previous detailed studies on the opinions of health care professionals regarding conscientious objection (CO) to induced abortion are relatively scarce and mostly the surveys concentrate on attitudes towards abortion and its indications *per se*. A U.S. survey in primary care revealed that 57% of respondents felt that physicians have sometimes obligations to provide services they believe to be morally wrong [3]. The controversial procedures that respondents objected to included abortions due to failed contraception (“social indications”) or due to Down syndrome (“medical condition of the fetus”). In the UK, 45% of medical students agreed that doctors should be entitled to object to any procedure for which they have a moral, cultural or religious disagreement [4]. Regarding abortion, objection to the procedure varied from 13% (abortion for a raped minor before 24 weeks) to 44% (abortion for congenital abnormalities after 24 weeks). It was reported in Mexico that CO was prevalent among newly-hired health care workers possibly due to insufficient knowledge about the legal and technical aspects of abortion, but unfortunately no exact figures on the prevalence were given [5]. In South Africa, 87% of medical students agreed that CO should be allowed to health care professionals [6]. A survey in the U.S. revealed that if a physician has a moral objection to procedures (such as abortions for gender selection), >90% were willing to refer patients to a colleague [7].

CO to participating in induced abortion is not present in the Finnish health care system or legislation unlike in many other European countries [8]. At present, 21 European Union member states grant CO by law. In Italy, 70% of OB/GYN and 50% of anesthesiologists have registered as professionals with CO [9]. Respective figures in favor of CO are 14% in Hong Kong (physicians of varied specialties), almost one third in the UK (OB/GYN trainees) and as high as 80% in Portugal (GYN). The willingness to provide abortion services depends on the clinical context and reason for abortion. For instance in Argentine, OB/GYN (>75%) were mostly willing to accept induced abortion in cases of severe fetal anomalies, maternal health threat and criminal conception despite their general opposition to abortion *per se*. While it has been considered important that CO should not cause any hindrance for women seeking appropriate services, in reality access to abortion has become more limited in several countries. This is exemplified by Austria, where there are regions with no providers of abortion [8,9]. For instance, the abortions in Salzburg area are conducted at one public hospital by providers who travel each week from Vienna [10]. In Italy, 69% of GYN practice CO based on the right to not perform activities that are “specific and necessary” to abortion [11]. The rule is ambiguous and it has caused at least one court case and problems to provide abortion care. As a result, many Italian women seek abortion abroad, such as in the UK or France.

In Finland, the possibility to CO has lately emerged in discussion [12]. Members of the government and the parliament have expressed strong demands to introduce CO in Finnish hospitals. While the discussion is lively, it mostly concentrates on CO as a dichotomy, it is either supported or rejected. For instance, Finnish GYN opposed the notion [13]. Although CO could ultimately be reduced to a simple choice between “CO and no CO”, we wished to include the option of “CO in some circumstances only and what would be its ramifications”. This means that while CO can be a yes–no dichotomy at the philosophical level, at the practical level of legislation it becomes a more complex issue, as all indications for legal abortion would not necessarily become indications for the CO of staff members.

Our aim was to study how Finnish medical and nursing students and professionals assess CO both *per se* (if it should be allowed or not) and as a complex process that includes various participants, indications and stages (what are the professions and instances for which CO could be allowed). We hypothesized that, as medical professionals and nurses have different educational backgrounds and they are responsible for different parts of the process, they would also show divergent opinions regarding indications for induced abortion and CO and reveal closer identification to issues related to their own profession. In a similar manner, we expected differences according to the stage of education and between students and professionals due to accumulating knowledge and experience on the subject.

## Methods

To study detailed opinions on CO, we formulated a survey that included questions as follows: general demographic data, defining the timeline of the embryo/fetus “becoming a person” during pregnancy, opinions on the definition of induced abortion, if CO should be introduced and if the respondent would personally wish to be allowed to use CO. In addition, the respondents answered questions about the adequacy of different convictions and which medical and legal indications should be included to CO (such as social indications *vs*. life-threating conditions; or different gestational ages, such as <12 weeks *vs*. 12–20 weeks). The respondents also chose, which of the professionals participating in the process should in their opinion be allowed CO if such legislation were to pass. Finally, some hypothetical situations caused by CO were evaluated. The comprehensive list of the questions can be found in Additional file 1.

We recruited medical and nursing students and professionals in the region of Northern Savo, Finland, at the Kuopio University Hospital district in 2013. The questionnaire was initially distributed to a pilot group of 5 medical and 5 nursing students to assess the structure and comprehensibility of the survey. This was followed by the actual study. The questionnaires were distributed during lecture hours and, for the professionals, by contact persons at wards and clinics. The first groups of the medical and nursing students were of the 1^st^ study year and the other student groups represented the last whole study year (5^th^-year medical students, University of Eastern Finland, School of Medicine; 4^th^-year nursing students, Savonia University of Applied Sciences, Schools of Health Care and Social Services and Health Care). The medical and nursing professionals represented various medical disciplines in hospitals and primary health care of the area. The respondents were explained the content of the study, answering was voluntary and the respondents answered to the questionnaire anonymously. No information that would have revealed the identity of the respondents was collected. Based on the regulations of the Finnish Advisory Board on Research Integrity [Ethical principles of research in the humanities and social and behavioural sciences and proposals for ethical review (http://www.tenk.fi/en/ethical-review-human-sciences)], no evaluation of an ethical board was necessary. A total of 240 medical students and professionals and 308 nursing students and professionals returned the questionnaire (response rate 66–100%). The questionnaires were returned directly to the researchers (SL and PR) or collected by a nominated person (*e.g*., of a hospital department) and returned to the researchers via conventional mail.

The answers of the different respondent groups were compared to each other with the χ^2^ test and, if the test criteria were not met, with the Fisher’s exact test (SPSS *v*19.0, IBM, Armonk, NY, USA). Differences in the age of the respondents were analyzed with the nonparametric Kruskal–Wallis analysis of variance (ANOVA). The medical and nursing groups were compared within the profession and, in addition, the answers of the medical respondents were compared to the corresponding group of nurses (*e.g*., 1^st^-year medical students *vs*. 1^st^-year nursing students). Regarding the medical respondents, the answers of men *vs*. women were also analyzed, but this was not possible for the nursing groups due to the low number of male respondents (5.3–13.0%). The p value <0.05 was considered statistically significant. The results are mostly presented as percentage of the respondents giving a particular response.

## Results

The comprehensive list of the questions and responses is available in Additional file 1. The demographics of the respondents can be also seen in Table 1. The percentage of the respondents in a permanent relationship or with children increased during the studies and the subsequent work experience. In a similar manner, personal experience of participating in abortions increased from 3.2 to 92.1% (medicine) and from 7.6 to 68.7% (nursing). The 5^th^-year medical students and professionals had significantly more often experience on the abortion process than the respective nursing respondents.

**Table 1:** **General demographic data of the respondents** (**%**/**mean** ± **SE**)

	**M 1** ^**st**^ **year**	**M 5** ^**th**^ **year**	**MD**	**N 1** ^**st**^ **year**	**N 4** ^**th**^ **year**	**Nurse**	**P value**
**Number of respondents**	93	71	76	92	85	131	
**Response rate (%)**	92	89	66	100	97	84	
**Sex**							<10^−5^
Male (%)	50.5	38.0	26.3	13.0	9.4	5.3	
Female (%)	49.5	62.0	73.7	87.0	90.6	94.7	
**Age** **(years)**	22 ± <1^A^	26 ± <1^B^	43 ± 1^C^	25 ± 1^A^	29 ± 1^B^	40 ± 1^C^	<10^−5^
**Work experience** **(years)**	N/A	N/A	18 ± 1	N/A	N/A	14 ± 1	
**Children**							
With children (%)	1.1	11.4	78.9	33.7	36.9	80.2	<10^−5^
**Marital status**							<10^−5^
Single (%)	78.5	52.1	10.5	44.6	21.2	12.2	
Marriage or cohabitation (%)	21.5	46.5	84.2	53.3	74.1	74.8	
Divorced or separated (%)	0.0	1.4	2.6	2.2	3.5	11.5	
Widow (%)	0.0	0.0	2.6	0.0	1.2	1.5	

*M* = medical student, *MD* = medical doctor, *N* = nursing student, *N*/*A* = not applicable. Means with no common letter differ at p < 10^−5^ within the medical or nursing respondents (Kruskal–Wallis ANOVA).

**Figure 1:**
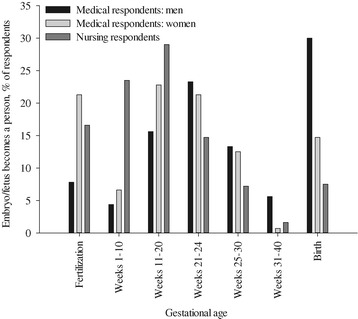
**The opinions of the respondents on the question**, **at which gestational age an embryo**/**fetus becomes a person.** The distribution of the male and female medical respondents (pooled according to gender) differs at p = 0.004 and the answers of the pooled nursing respondents differ from those of the medical respondents at p < 10^−5^ (Fisher’s exact test). Regarding the nursing respondents, no analysis between sexes was feasible due to the small number of men.

Opinions on the definition of induced abortion varied especially among the 1^st^-year students. The medical students assessed more often than the nursing students that intrauterine devices (IUD; 15.1 *vs*. 5.5%) and emergency contraception (34.4 *vs*. 19.8%) could also be classified as induced abortion. The difference about the status of IUD also persisted in the later-stage medical and nursing students (26.8 *vs*. 7.1%) but disappeared among the professionals. Among those who considered IUD a form of induced abortion, 28.6% of the nurses *vs*. 15.4% of the medical professionals would have wanted to gain CO and 42.9 *vs*. 30.8% were willing to grant CO to others. There were no differences compared to those who did not interpret IUD as abortion or between the professions. Regarding the timeline of “becoming a person” (“at which gestational age does an embryo/fetus become a person?” in the questionnaire), the nursing respondents mostly chose gestational weeks 0–24. Among the medical respondents, the women displayed two peaks, immediately at fertilization and between gestational weeks 11–24, while the opinions of the men were partly different (Fisher’s exact test, p = 0.004; Figure 1). A major part of them considered gestational weeks 11–30 to be crucial in this aspect, but 22–50% considered birth to be the determining point of time.

Personal volition to CO was relatively low, 3.5 (4^th^-year nursing students)–14.1% (5^th^-year medical students), while the willingness to allow CO was at a higher level, 10.6 (4^th^-year nursing students)–34.2% (medical professionals). Generally, the medical professionals were more prone to support CO than the nurses. Most students and professionals considered that they would be able to work in a team including persons with CO, 62.4 (1^st^-year medical students)–77.2% (1^st^-year nursing students). The 1^st^-year nursing students and the nursing professionals were the most willing to work with persons with CO. When assessing how the conviction for CO should be evaluated, the respondents considered either self-reported Christian (15.5–26.2%), another religious (15.5–27.7%) or ethical conviction (22.5–34.8%) or a simple statement without justification (17.4–36.2%) to be adequate. However, approximately one half (48.0–50.7%) of the medical students and professionals responded that they would consider no conviction to be adequate for CO (*vs*. 30.1–43.5% of the nursing students and professionals), which could indicate somewhat self-contradictory replies among the respondents. Furthermore, the majority of all respondents (57.9–72.8%) assessed that CO would cause conflicts at work communities.

Regarding the abortion process, very few respondents would include primary health care nursing, gestational age determination or post-abortion follow-up/contraceptive advice to CO. The medical students and professionals would more often include primary health care physicians referring patients to abortions and prescribing abortion-inducing pharmaceuticals to CO compared to the nursing students and nurses. The most common procedure that the respondents would include to CO was performing vacuum suction for abortion (31.0–45.7% of the medical students and professionals, 41.7–60.2% of the nursing students and professionals). A difference between the professions was observed: the nursing respondents were more willing to grant CO for vacuum suction (47.7%) than for medical abortions (15.7%; all nursing respondents pooled, χ^2^ test, p < 0.001) or referrals (9.1%; χ^2^ test, p < 0.001). In contrast, the medical respondents treated prescription and vacuum suction in a more uniform manner (31.2 *vs*. 39.7%, χ^2^ test, p = 0.068). Acceptable reasons for CO according to the respondents were social indications of the pregnant woman (18.3–22.7% of the medical students and professionals *vs*. 8.6–11.0% of the nursing students and nurses) and abortions performed during weeks 20–24 of gestation (12.7–21.3 *vs*. 23.5–43.9%) and during weeks 12–20 (11.3–20.0 *vs*. 9.9–19.5%). The medical professionals would include social indications to CO more often than the nurses (22.7 *vs*. 10.9%). Indications that a clear majority (generally >90% with few exceptions) would not include to CO were criminal cases (*i.e*., rape or incest), life-threatening or serious medical conditions of the woman or the fetus and the young age (<17 years) of the woman. Among all respondents, 32.9–62.0% considered that there would be no acceptable indications for CO.

When considering the right to CO based on professional titles, the medical and nursing respondents most often included an OB/GYN performing vacuum suction (16.9–34.8%) with no differences between the professional groups. The nursing students were less likely to allow an OB/GYN the right to CO regarding prescriptions than the medical students (8.4–14.6% *vs*. 25.7–29.3%). Regarding the other medical professions, the 5^th^-year medical students would have granted CO more often to radiologists compared to the respective nursing students (16.9 *vs*. 6.0%), while the respondents had similar opinions on the potential right to CO by anesthesiologists (9.6–22.4%). Eagerness to include various personnel assisting an OB/GYN (nurses at prenatal/outpatient care, midwives, operating room nurses, supply technicians) did not differ according to the respondent group with values between 8.4–28.3%. Only a few respondents (1.2–6.3%) were willing to apply CO during outpatient follow-up (contraception consultation) after induced abortion.

The respondents were also questioned about potential implications of CO to the training of OB/GYN/nurses. The majority of the medical respondents (60.8–75.0%) considered that, during training, the procedures of abortion must be practiced in order to be able to perform during emergencies, while a minority (13.2–23.0%) would have been satisfied with observation without actual practice. The nursing students and professionals were questioned about their training that would include observing and assisting in induced abortions. They considered it important to either observe (37.5–47.6%) or to participate in abortions (26.2–45.5%) during education. Regarding emergencies and periods with low number of staff (vacations, nighttime), the most common reply of the respondents (38.9–58.1%) was that the conviction should be secondary and the person with CO should perform the required procedures. Relatively many considered direct orders by a supervisor (18.1–30.1%) as adequate grounds to discard CO or cooperation between hospitals (17.2–38.9%) to be an acceptable alternative. Few respondents would have chosen CO instead of the abortion procedure in case of a medical emergency (1.1–4.2%). Quite similar responses were obtained regarding a scenario, where an anesthesiologist/operating room nurse unwilling to participate in abortion would be the only specialist available: only 0.0–7.2% of the respondents considered that CO would be the alternative of choice.

Among the 1^st^-year medical students, the men considered significantly more often than the women that contraceptive pills and IUD would be forms of induced abortion (23.4 *vs*. 6.5% for both). The men were more prone than the women (59.6 *vs*. 39.1%) to refuse the right to CO. Regarding the indications, the men considered more often than the women (13.0 *vs*. 0.0%) that life-threatening conditions of the pregnant woman should be included to CO. Among the 5^th^-year medical students, the men considered emergency contraception to be a form of abortion more often than the women (59.3 *vs*. 31.8%). The women included more often referral by a general practitioner (29.5 *vs*. 7.4%) to CO. Among all medical respondents, the women were more willing to comprise vacuum suction (46.6 *vs*. 29.3%) and referrals in primary health care to CO (26.0 *vs*. 14.1%). The men were more accepting to CO in cases of a life-threatening emergency (12.0 *vs*. 2.7%; p = 0.006) or a serious medical condition of the woman (12.0 *vs*. 4.8%; p = 0.048) or a life-threatening condition of the fetus (12.0 *vs*. 4.1%; p = 0.036).

## Discussion

Despite lively discussion, there is no Finnish legislation on the possibility of CO [8]. Generally, the debate has focused on a simplified yes–no axis without considering the potential ramifications of CO [12]. We wished to augment the discussion by including in our survey relevant points that should be considered if CO were to become an accepted alternative also in Finland. Among these aspects are the assessment of the specific tasks and professions that would be included to CO, the indications for the termination of pregnancy and the ultimate effects CO would have on OB/GYN or nursing education and at the workplace. To our knowledge, this is the first study to assess the question of CO in a more complex manner that, however, reflects the real-life situation that has to be dealt with at the health care services. While the survey was conducted on only one geographical area, we managed to obtain a fairly large number of respondents. Thus, we think that the survey is valid within the district and, due to the small Finnish population, also within our country. Still, there are some limitations, as the number of male respondents within the nursing groups was too small for meaningful comparisons. In addition, the results of the present survey cannot be directly generalized across national borders. Due to this, we propose that similar surveys including in detail the legislative patterns of each country regarding induced abortion and CO should be conducted, and the present questionnaire can be fine-tuned to assess the situation elsewhere.

Considering CO, major world religions oppose induced termination of pregnancy [14]. Principal Christian denominations either condemn all indications for abortion or reserve the right for termination of pregnancy only to emergencies (such as the Catholic Church). However, some protestant (Lutheran) denominations, while in principle not accepting abortion, show understanding to the complexity of the issue and state that there are life situations that force us to choose between the greater and lesser evils. In Judaism, many scholars consider that all cases should be assessed individually with no generalized commands on abortion. In the Torah (Exodus 21: 22–23), there are passages that value the life of a pregnant woman higher than that of the fetus.

The debate on abortion focuses on two complex ethical issues as follows: *i*) is the embryo/fetus at all or at some stages of pregnancy unequivocally entitled to protection of its life and *ii*) is the pregnant woman obliged to allow the embryo/fetus to use her body on some or all occasions [15]. Both issues are unresolved and it has been suggested that because of the inability of obtaining an indisputable result, the freedom of conscience in liberal societies should include regulated abortion. This would lead to legal abortion being available but, on the other hand, the potential question of the right to express CO emerges. In fact, CO is a situation, where a staff member puts his/her rights and morals before the rights of a patient who wishes to receive a legal procedure [16], and some authors take this as an undefendable position stating that the first obligation should be to the patients and not to the staff members themselves [10]. Thus, it has been suggested that the objectors should be obliged to justify their position [17]. From the viewpoint of the work community this means “proving genuineness”, as it is assumed that free access to CO would attract free-riders that would simply wish to avoid unpleasant duties.

It has also been claimed that the taking part in duties that the objector finds immoral would be unreasonable only if the moral harm for the objector would be greater than the harm suffered by the patient with a more limited access to abortion [18]. However, the evaluation of genuineness is difficult and complex. The objection can rest on empirically (*e.g*., inadequate knowledge on embryonic development and fetal perception of pain makes the objector to interpret reflex fetal movement as a response to pain [19]) or morally weak bases (discriminatory beliefs based on morals and/or religion, such as women being irresponsible when not using adequate contraception [17]). In these cases, the claim for CO may be genuine but if it is based on ignorance or discriminatory beliefs it should not necessarily be supported. One ought to also consider the voluntary nature of being involved in health care and choosing one’s profession. The responsibility of the objector is, in fact, greater than that of the patient, as there was no obligation for the person demanding CO to choose a particular profession or a specialty. Kantymir and McLeod [17] suggest that the objection (not performing an abortion) should be reasonable, *i.e*., the beliefs motivating the objection should be as likely or more likely true than the beliefs that support the service (performing abortion) the objector finds offensive, or the objection should be genuine. In addition, they suggest that a genuine objection should satisfy particular criteria: patients’ access to care should be provided respectfully and without delay, and CO should not be based on, *e.g*., discriminatory reasons. This would require assessment of the moral position of the person requesting CO and the objectors would still have duties to patients by ensuring adequate care (*e.g*., referring to willing professionals).

In addition to general religious and philosophical considerations, CO has been assessed by international medical associations [20,21]. The consensus is that professionals should have a right to CO without this causing any discrimination against them. However, CO should be considered secondary to the duty to treat patients in emergencies and to refer patients to willing providers without delay. These issues were, in our opinion, quite well covered by our survey. Considering our results, the respondents would be stricter than these recommendations and 49.5–69.4% would not support the right to CO, possibly advocating the position of career and specialty choices being voluntary as also stated by Finnish GYN [13]. However, when assessing further the possible acceptable justifications for CO, approximately one third would accept CO based on a simple statement. Still, only a minority would seek CO for themselves (3.5–14.1%). Based on the previous discussion, CO based on a simple statement is the position that would allure potential free-riders as well as objectors with CO based on misconceptions and/or discriminatory reasons. The respondents seemed to have considered this possibility as a clear majority of them (57.9–72.8%) anticipated problems at the work communities if CO is applied, while most, however, stated that they would be able to work in a team with persons demanding CO (62.4–77.2%). If legislation for CO is forwarded, it would be essential to assess the possibility of social conflicts. Regarding the respondents, their views about CO seemed to be quite divided and inconsistent further emphasizing the need to discussing the issue in all its complexity (regulated CO) and not as a dilemma of “CO–no CO”. The criterion of providing adequate care if CO is legalized was also partly assessed by our survey, as a clear majority of the respondents would allow CO to be overridden in emergencies or by a direct order of the employer. We noticed that ≤7.2% of the respondents would allow CO in a medical emergency. This also has potential ramifications to choosing one’s profession. Most respondents considered either observing (13.2–47.6%) or actually practicing/assisting in the abortion procedure (26.2–75.0%) to be vital when training to be an OB/GYN or an operating room nurse/midwife.

When considering the global situation, the legal right to safe abortion is far from self-evident. Prohibition of abortion or strict laws regulating the access to abortion apart from medical emergencies do not correlate with lower numbers of abortions in these countries [22]. In fact, it has been assessed that the proportion of unsafe abortions is higher with restrictive abortion laws causing significant morbidity and mortality. Thus, if the proponents of CO wished not only to provide personal gratification for those unwilling to participate in abortions but also, for instance, to decrease the general number of abortions in a country, a strategy that makes access to safe abortion more difficult can be unsuccessful. This raises the question if the medical/nursing professionals are willing to put their personal convictions ahead of patients’ rights, as has been the case, *e.g*., in Austria and Italy, where the access to safe abortion has become more difficult [8,11].

Legal abortions in Finland are mostly performed medically (87% [2]) without surgical intervention but if the final outcome is considered, the two methods for the termination of pregnancy have obviously the same result. Still, the nursing respondents were more willing to grant CO for surgical procedures than to inducing abortions with medication (*e.g*., 60.2 *vs*. 19.3% for the 1^st^-year nursing respondents). This could reflect a difference in the education of medical doctors and nurses, as the medical respondents displayed no significant differences in their responses regarding medical or surgical abortions. This suggests that the actual outcome (death of the embryo/fetus) is not the only aspect considered. The procedure of vacuum suction could be perceived more drastic and unpleasant from the staff’s point of view. In our opinion, this is an issue that requires further assessment, as it indicates that the personal comfort of the staff could in these cases be placed before the care of the patient. This attitude could be problematic regarding the nursing profession, as it also indicates that the respondents have not necessarily considered the similar outcome quite rationally.

Similarly, the indications for abortion were considered in a diverse manner by the respondents. Only a minority (2.5–8.7%) considered that CO should be applied in cases of criminally conceived pregnancy and similar numbers were obtained for medical emergencies. Especially the medical respondents (18.3–22.7%) would allow CO for abortions due to social indications. If CO is granted but restricted according to indication, this could lead to situations where the professional secrecy is jeopardized. In a hypothetical scenario, a staff member refuses to participate in abortions except in cases of crime. Thus, he/she would automatically know that when participating in the process of abortion, the patient would be a victim of rape or incest, although the patient could wish to keep the indications of her abortion undisclosed.

Among the medical respondents, significantly more of the men (12%) were willing to allow CO in cases, where the termination of pregnancy would be indicated due to a life-threatening situation of the pregnant woman. In fact, very few of the female medical respondents (2.7%) would have considered CO an option in those circumstances. It is tempting to hypothesize that this could have been caused by stronger identification of the female respondents to the situation of the pregnant woman. Some previous data could support this speculative explanation. In a Chinese study among medical students, women exhibited significantly higher empathy scores compared to men [23] and a similar trend was observed for physicians in the U.S. [24]. Unfortunately, no empathy scoring studies related directly to the issue of abortion were available, but there remains the alternative that women would be prone to feeling more empathy towards the situation of the pregnant woman and men towards the embryo/fetus. Although this gender-related discrepancy cannot be explained by the present survey, we feel that this tendency of disregarding life-threatening situations in favor of personal conviction is slightly alarming, although only a minority of the male respondents was of this opinion.

Another gender-related difference among the medical respondents was how they perceived which gestational age would be the threshold of “becoming a person”, *i.e*., the point of time when the fetus could be perceived as a being with at least some moral status. The men placed the point of time significantly more often at birth than the women. In addition, the medical students and professionals also considered birth to be the crucial point of time more often than the nursing respondents. The question is difficult to answer both scientifically and based on possible religious convictions. Many religions consider fertilization to be the start point of human life [14], but this opinion was shared only by a minority (13.6–19.4%) in our study. The respondents usually chose gestational ages between weeks 0–24 (nursing respondents) or 11–30 (medical respondents) suggesting that their opinions could be also influenced by scientific data rather than religious doctrines. From the scientific point of view, it has been considered crucial to assess when the sensory apparatus of the fetus becomes mature enough for the perception of pain, which would have significance for the time limits of abortion. According to available data, Lee et al. [25] conclude that the conscious perception of pain only develops during gestational weeks 29–30. While the survey cannot explain the difference between the two professions, it is conceivable that the different education and the more detailed studies on human embryology by the medical students could play a role here [26,27]. A misconception about the nature of IUD was an item we looked into in more detail. However, the willingness to support CO did not vary according to the false belief that IUD would be abortifacient [28].

Although the question about CO is often simplified to a dilemma (yes–no), legislation requires an assessment of diverse aspects and ramifications of CO and the multi-professional process of abortion. The principal points to consider are the stage of pregnancy, indications for abortion, professions, procedures and the training of future specialists in nursing and medicine. As stated previously [8,16,17], a crucial issue is to ensure that legal abortion care can be provided even in the case CO is allowed. Unfortunately, access to safe abortion has become more limited in Europe [8]. While a large part of our respondents seemed to acknowledge this issue by accepting no CO in cases of medical emergencies, limiting CO to social indications could have ramifications in patient confidentiality and professional secrecy. In addition, there are potential deficits in the education of professionals considering fetal development and the actual state of abortions being mostly medical and more rarely surgical. In fact, the curricula of nurses and midwives at the Savonia University of Applied Sciences [27] have no credits allocated for embryology *per se* (*vs*. 3.0 credits for medical students [26]). The opinions seemed quite divided, as approximately one half–two thirds (49.5–69.4%) of our respondents would not support CO. To promote rational discussion on the subject, professionals, politicians and the general public should be made more aware of the complexity of the issue and the possible effects of CO on patient care based, *e.g*., on the experiences of countries where CO is practiced to ensure also the continued promotion and protection of women’s health [29].

## Conclusions

The opinions of the respondents gave a mixed response regarding support to CO. While the respondents mostly seemed to consider the continuation of adequate services important if CO is introduced, the viewpoint was often focused on the staff and surgical abortion procedure instead of the patients. The issue proved to be complex, which should be taken into consideration for legislation.

## Additional file

Additional file 1:
**Questions and answers to the questionnaire on CO to induced abortion.** The values are percentages unless otherwise indicated.

## Abbreviations

ANOVA: Analysis of variance
CO: Conscientious objection
IUD: Intrauterine device
OB/GYN: Obstetrician/gynecologist
