# Supplementary material for: Opinions on conscientious objection to induced abortion among Finnish medical and nursing students and professionals
Source: BMC Med Ethics. 2015 Mar 25;16:17. doi: 10.1186/s12910-015-0012-1 (PMC4376492; doi:10.1186/s12910-015-0012-1)
Supplement: Additional file 1: — Questions and answers to the questionnaire on CO to induced abortion. The values are percentages unless otherwise indicated. [file 12910_2015_12_MOESM1_ESM.doc]

**Additional file 1.** Questions and answers to the questionnaire on CO to induced abortion. The values are percentages unless otherwise indicated.

|  | **M 1^st^ yr** | **M 5^th^ yr** | **MD** | **N 1^st^ yr** | **N 4^th^ yr** | **Nurse** | **P value** |
| --- | --- | --- | --- | --- | --- | --- | --- |
| **Number of respondents** | 93 | 71 | 76 | 92 | 85 | 131 |  |
| **1. Sex** |  |  |  |  |  |  | <10^–5^ |
| Male | 50.5 | 38.0 | 26.3 | 13.0 | 9.4 | 5.3 |  |
| Female | 49.5 | 62.0 | 73.7 | 87.0 | 90.6 | 94.7 |  |
| **2. Age (years; mean ± SE)** | 22 ± <1^A^ | 26 ± <1^B^ | 43 ± 1^C^ | 25 ± 1^A^ | 29 ± 1^B^ | 40 ± 1^C^ | <10^–5^ |
| **3. Work experience (years; mean ± SE)** | N/A | N/A | 18 ± 1 | N/A | N/A | 14 ± 1 |  |
| **4. Do you have children?** |  |  |  |  |  |  |  |
| Yes | 1.1 | 11.4 | 78.9 | 33.7 | 36.9 | 80.2 | <10^–5^ |
| **5. Marital status** |  |  |  |  |  |  | <10^–5^ |
| Single | 78.5 | 52.1 | 10.5 | 44.6 | 21.2 | 12.2 |  |
| Marriage or cohabitation | 21.5 | 46.5 | 84.2 | 53.3 | 74.1 | 74.8 |  |
| Divorced or separated | 0.0 | 1.4 | 2.6 | 2.2 | 3.5 | 11.5 |  |
| Widow | 0.0 | 0.0 | 2.6 | 0.0 | 1.2 | 1.5 |  |
| **6. Which of the following are methods for induced abortion? *Choose as many alternatives as you wish.*** |  |  |  |  |  |  |  |
| Abortion | 94.6 | 98.6 | 89.5 | 93.4 | 95.3 | 92.4 | 0.292 |
| Emergency contraception | 34.4^b^ | 42.3 | 27.6 | 19.8^a^ | 32.9 | 39.7 | 0.018 |
| Contraceptive pills | 15.1 | 7.0 | 13.2 | 7.7 | 5.9 | 9.2 | 0.258 |
| IUD | 15.1^b^ | 26.8^b^ | 17.1 | 5.5^a^ | 7.1^a^ | 10.7 | 0.003 |
| **7. At which gestational age does an embryo/fetus become a person?** |  | * | * |  | * | * | <10^–5^ |
| Immediately at fertilization | 13.6 | 19.4 | 15.5 | 15.4 | 16.9 | 17.5 |  |
| At 1–10 weeks | 6.8 | 1.5 | 8.5 | 25.3 | 16.9 | 26.2 |  |
| At 11–20 weeks | 21.6 | 13.4 | 23.9 | 30.8 | 25.3 | 30.2 |  |
| At 21–24 weeks | 21.6 | 23.9 | 21.1 | 15.4 | 15.7 | 14.3 |  |
| At 25–30 weeks | 13.6 | 10.4 | 14.1 | 5.5 | 13.3 | 4.0 |  |
| At 31–40 weeks | 5.7 | 0.0 | 1.4 | 2.2 | 2.4 | 0.8 |  |
| Immediately at birth | 17.0 | 31.3 | 15.5 | 5.5 | 9.6 | 7.1 |  |
| **8. Have you participated at any stage of the process of abortion during your studies or working life? (referral, procedure, follow-up, observation of the procedure)** |  |  | <10^–5^ |  |  | <10^–5^ |  |
| Yes | 3.2^aA^ | 78.9^bB^ | 92.1^bC^ | 7.6^bA^ | 51.8^aB^ | 68.7^aC^ |  |
| **9. Would you support the right for CO to participating in abortions?** |  |  |  |  |  |  | 0.0037 |
| Yes | 24.7 | 22.5 | 34.2^b^ | 21.7 | 10.6 | 21.4^a^ |  |
| No | 49.5 | 60.6 | 56.6^b^ | 52.2 | 69.4 | 49.6^a^ |  |
| Does not know | 25.8 | 16.9 | 9.2^a^ | 26.1 | 20.0 | 29.0^b^ |  |
| **10. In your profession, would you like to be able to refuse to participate in abortions?** |  |  |  |  |  |  | 0.148 |
| Yes | 5.4 | 14.1^b^ | 13.5 | 9.8 | 3.5^a^ | 9.2 |  |
| No | 77.4 | 77.5^a^ | 71.6 | 68.5 | 82.4^b^ | 72.3 |  |
| Does not know | 17.2 | 8.5^a^ | 14.9 | 21.7 | 14.1^b^ | 18.5 |  |
| **11. Would you be able to work in a team including someone who refuses to participate in abortions?** |  |  |  |  |  |  | 0.031 |
| Yes | 62.4^a^ | 69.0 | 69.3^a^ | 77.2^b^ | 65.9 | 74.0^b^ |  |
| No | 14.0^b^ | 15.5 | 18.7^b^ | 2.2^a^ | 12.9 | 7.6^a^ |  |
| Does not know | 23.7^b^ | 15.5 | 12.0^a^ | 20.7^a^ | 21.2 | 18.3^b^ |  |
| **12. What would you consider an adequate conviction, based on which one could refuse to participate in abortions? *Choose as many alternatives as you wish.*** |  |  |  |  |  |  |  |
| Christian conviction assessed by a psychologist | 18.5 | 5.6 | 5.3 | 20.2 | 14.1 | 11.5 | 0.014 |
| Another religious conviction assessed by a psychologist | 18.5 | 5.6^a^ | 5.3 | 19.1 | 16.5^b^ | 12.3 | 0.020 |
| Ethical conviction assessed by a psychologist | 23.9 | 7.0 | 5.3 | 29.2 | 15.3 | 13.1 | 5.2×10^–5^ |
| Self-reported Christian conviction | 25.0 | 15.5 | 24.0 | 21.3 | 22.4 | 26.2 | 0.638 |
| Self-reported another religious conviction | 23.9 | 15.5 | 24.0 | 19.1 | 21.2 | 27.7 | 0.430 |
| Self-reported ethical conviction | 34.8 | 22.5 | 34.7 | 34.8 | 27.1 | 31.5 | 0.455 |
| A simple statement with no justification | 17.4^a^ | 35.2^b^ | 33.3 | 31.5^b^ | 20.0^a^ | 36.2 | 0.011 |
| None of the above is adequate | 48.9^b^ | 50.7 | 48.0 | 30.1^a^ | 43.5 | 37.6 | 0.066 |
|  |  |  |  |  |  |  |  |
| **Multiplicative effects** |  |  |  |  |  |  |  |
| **1. Which parts of the abortion processes should CO include? *Choose as many alternatives as you wish.*** |  |  |  |  |  |  |  |
| Guidance to abortion in primary health care [nurse] | 6.5 | 11.3 | 13.3 | 4.8 | 3.6 | 7.1 | 0.158 |
| [MD in] primary health care referring the patient to abortion | 13.0 | 21.1^b^ | 32.0^b^ | 9.6 | 4.8^a^ | 11.1^a^ | 1.2×10^–5^ |
| [MD] determining gestational age for abortion | 3.3 | 7.0 | 10.7 | 6.0 | 3.6 | 4.0 | 0.318 |
| [MD] writing the prescription to medical abortion | 34.1^b^ | 26.8^b^ | 32.0^b^ | 19.3^a^ | 9.5^a^ | 17.5^a^ | 0.001 |
| [MD] performing abortion with vacuum suction | 45.7 | 31.0 | 41.3 | 60.2 | 41.7 | 42.9 | 0.014 |
| [Nurse] assisting in vacuum suction | N/A | N/A | N/A | 47.0 | 35.7 | 41.3 | 0.335 |
| [MD or nurse] follow-up after abortion | 3.3 | 2.8 | 1.3 | 7.2 | 0.0 | 2.4 | 0.123 |
| [MD or nurse] contraceptive advice after abortion | 1.1 | 1.4 | 1.3 | 4.8 | 0.0 | 2.4 | 0.365 |
| Other part of the process | 1.1 | 0.0 | 1.3 | 0.0 | 0.0 | 2.4 | 0.391 |
| None of the above should be included | 50.0 | 63.4 | 53.3 | 37.5 | 53.6 | 48.4 | 0.088 |
| **2. What are the situations, when the personnel should be allowed to refuse to participate in abortions?** ***Choose as many alternatives as you wish.*** |  |  |  |  |  |  |  |
| The child was conceived due to crime (*i.e*., rape or incest) | 8.7 | 7.0 | 5.3 | 4.9 | 2.5 | 3.1 | 0.147 |
| Life-threatening condition of the woman | 6.5 | 8.5 | 4.0 | 1.2 | 2.5 | 3.9 | 0.269 |
| Serious medical condition of the woman | 7.6 | 8.5 | 6.7 | 3.7 | 2.5 | 3.9 | 0.425 |
| Life-threatening condition of the fetus | 7.6 | 8.5 | 5.3 | 4.9 | 3.7 | 4.7 | 0.773 |
| Serious medical condition of the fetus | 12.0 | 8.5 | 9.3 | 4.9 | 4.9 | 5.5 | 0.358 |
| Twin-to-twin transfusion syndrome | 9.8 | 7.0 | 5.3 | 7.3 | 3.7 | 3.1 | 0.350 |
| Social indications of the woman | 21.7 | 18.3 | 22.7^b^ | 11.0 | 8.6 | 10.9^a^ | 0.028 |
| The woman is less than 17 years of age | 12.0 | 8.5 | 9.3 | 8.5 | 3.7 | 8.6 | 0.568 |
| The woman has ≥ 4 previous children | 16.3 | 12.7 | 14.7 | 12.2 | 6.2 | 9.4 | 0.340 |
| All abortions during gestational weeks 20–24 | 20.7^a^ | 12.7 | 21.3 | 43.9^b^ | 23.5 | 27.3 | 3.3×10^–4^ |
| All abortions during gestational weeks 12–20 | 12.0 | 11.3 | 20.0 | 19.5 | 9.9 | 14.1 | 0.304 |
| All abortions also before gestational week 12 | 9.8 | 15.5 | 14.7 | 11.0 | 7.4 | 8.6 | 0.478 |
| None of the above is adequate for CO | 50.0^b^ | 62.0 | 60.0 | 32.9^a^ | 50.6 | 52.4 | 0.008 |
| **3. Which one of the persons involved in abortions should have the right to CO? *Choose as many alternatives as you wish.*** |  |  |  |  |  |  |  |
| Nurse at a prenatal clinic | 20.7 | 18.3 | 22.4 | 18.3 | 15.7 | 19.7 | 0.926 |
| MD at primary health care clinic | 26.1 | 26.8^b^ | 34.2 | 17.1 | 10.8^a^ | 22.0 | 0.0095 |
| Nurse at the outpatient clinic of a hospital | 22.8 | 18.3 | 25.0 | 26.8 | 14.5 | 28.3 | 0.209 |
| OB/GYN making a prescription for medical abortion | 29.3^b^ | 25.7^b^ | 31.6 | 14.6^a^ | 8.4^a^ | 22.8 | 0.002 |
| OB/GYN performing vacuum suction | 34.8 | 25.4 | 34.2 | 31.7 | 16.9 | 29.9 | 0.100 |
| Midwife | 22.8 | 16.9 | 15.8 | 20.7 | 13.3 | 22.8 | 0.359 |
| Radiologist | 15.2 | 16.9^b^ | 15.8 | 20.7 | 6.0^a^ | 18.9 | 0.133 |
| Anesthesiologist | 17.4 | 15.5 | 22.4 | 17.1 | 9.6 | 18.9 | 0.392 |
| Operating room nurse | 19.6 | 16.9 | 22.7 | 20.7 | 15.7 | 23.6 | 0.733 |
| Supply technician | 18.5 | 15.5 | 15.8 | 23.2 | 8.4 | 17.3 | 0.225 |
| Ward domestic | 18.5^a^ | 19.7 | 11.8 | 31.7^b^ | 14.5 | 20.5 | 0.034 |
| Contraception consultant [MD or nurse] during follow-up | 5.4 | 2.8 | 5.3 | 4.9 | 1.2 | 6.3 | 0.548 |
| Another professional | 2.2 | 0.0 | 1.3 | 2.4 | 1.2 | 5.5 | 0.256 |
| None of the above-mentioned should be given the right for CO | 43.5 | 57.7 | 57.9 | 38.9 | 55.4 | 51.6 | 0.109 |
| **4. If the right to CO to abortion is realized, how should the training for future OB/GYN or nurses/midwives be arranged? *Choose only one alternative.*** |  |  | 0.011 |  |  | 0.119 |  |
| It should be possible to specialize to be an OB/GYN or an operating room nurse/midwife without participating in abortions even during medical emergencies. | 1.1 | 8.8 | 10.8 | 3.4 | 6.1 | 4.8 |  |
| Theoretical knowledge about abortions and skills in conventional abrations are adequate. | 1.1 | 7.4 | 5.4 | 13.6 | 14.6 | 21.4 |  |
| During training, one must observe abortion procedures in order to be able to perform them in medical emergencies. | 22.8 | 13.2 | 23.0 | 37.5 | 40.2 | 47.6 |  |
| During training, one must practice/assist in abortion procedures in order to be able to perform them in medical emergencies. | 75.0 | 70.6 | 60.8 | 45.5 | 39.0 | 26.2 |  |
| **5. If the right to CO to abortion is realized, do you think it will cause conflicts at work communities?** |  |  |  |  |  |  | 0.105 |
| Yes | 64.5 | 60.6 | 57.9 | 72.8 | 69.4 | 64.1 |  |
| No | 10.8 | 8.5 | 22.4 | 9.8 | 7.1 | 11.5 |  |
| Does not know | 24.7 | 31.0 | 19.7 | 17.4 | 23.5 | 24.4 |  |
| **If the right to CO to abortion is realized, how would you deal with the following scenarios? *Choose only one alternative.*** |  |  |  |  |  |  |  |
|  |
| **1. A small community does not have a MD (because of CO) willing to refer abortion patients to hospitals/a nurse referring an abortion-seeking woman to a MD.** |  |  | 0.021 |  |  | 0.938 |  |
|
| In an emergency, the professional lets his/her conviction to be overridden and takes part in the abortion process. | 58.1 | 42.0 | 38.9 | 47.8 | 43.4 | 41.1 |  |
|
| The employer or the manager has the right to order the professional to take part in the abortion process despite of any conviction if the situation requires that. | 23.7 | 20.3 | 18.1 | 23.3 | 30.1 | 29.5 |  |
| The issue can be dealt with cooperation between hospitals and primary health care units. | 17.2 | 34.8 | 38.9 | 26.7 | 24.1 | 26.4 |  |
| It is primary to respect the conviction of the professional and to accept potential problems. | 1.1 | 2.9 | 4.2 | 2.2 | 2.4 | 3.1 |  |
| **2. In a small hospital no anesthesiologist/operating room nurse is willing to participate in abortion because of CO.** |  |  | 0.003 |  |  | 0.904 |  |
| In an emergency, the professional lets his/her conviction to be overridden and takes part in the abortion process. | 63.4 | 53.6 | 36.2 | 46.1 | 53.0 | 43.8 |  |
|
| The employer or the manager has the right to order the professional to take part in the abortion process despite of any conviction if the situation requires that. | 25.8 | 24.6 | 29.0 | 23.6 | 22.9 | 26.6 |  |
| The issue can be dealt with cooperation between hospitals and primary health care units. | 10.8 | 18.8 | 27.5 | 28.1 | 22.9 | 27.3 |  |
| It is primary to respect the conviction of the professional and to accept potential problems. | 0.0 | 2.9 | 7.2 | 2.2 | 1.2 | 2.3 |  |

CO=conscientious objection, IUD=intrauterine device, M=medical student, MD=medical doctor, N=nursing student, N/A=not applicable, OB/GYN=obstetrician/gynecologist. Lower-case superscript letters indicate differences between the medical and nursing students/professionals of the same stage. Capital superscript letters indicate differences within the medical or nursing respondents. *=Difference in the distribution of answers in the columns allocated for a particular question between the medical and nursing students/professionals of the same stage. χ^2^-test/Fisher’s exact test/Kruskal–Wallis ANOVA.
